# Changes in End-of-Life Quality in Patients With Terminal Cancer During Their Last 6 Months of Life: A Longitudinal Study

**DOI:** 10.1097/jnr.0000000000000711

**Published:** 2025-11-12

**Authors:** Chen Hsiu Chen, San Chi Chen, Hsing Jung Lee

**Affiliations:** 1 School of Nursing, National Taipei University of Nursing and Health Sciences, Taipei, Taiwan, ROC; 2 Department of Oncology, Division of Medical Oncology, Taipei Veterans General Hospital, Taipei, Taiwan, ROC; 3 Department of Nursing, Taipei Veterans General Hospital, Taipei, Taiwan, ROC

**Keywords:** terminal care, Quality of Dying and Death Questionnaire, longitudinal studies, patient satisfaction, neoplasm

## Abstract

**Background:**

Optimizing end-of-life (EOL) quality is crucial for patients with cancer. However, most studies in the literature explore EOL quality in the last month of life for these patients and use retrospective investigations based on information from bereaved family caregivers.

**Purpose:**

In this study, the authors aimed to investigate the underexplored changes in EOL quality perceived by patients with cancer during their last 6 months of life.

**Methods:**

A longitudinal approach was used to investigate a convenience sample of 229 patients recruited at 2 medical centers in northern Taiwan from January 2020 to July 2023. The Quality of Dying and Death Questionnaire was used to measure EOL quality, and changes in EOL quality were analyzed using multivariate generalized estimating equations.

**Results:**

As the participants approached their terminal stage, EOL-quality total scores gradually declined (from 71.41±7.56 to 65.27±10.04), and the proportion of scores classified as “good to almost perfect” (i.e., ≥70) declined from 68.6% to 34.6% over the same period. After controlling for covariates, EOL-quality subdomains and total scores exhibited variable trends, with total EOL-quality scores significantly lower in the last 2 months than in the 91–180 days before death. In terms of the subdomains, “symptoms and personal control” had the lowest average scores and substantially decreased over the last 3 months, while scores for “death preparation” (the second-lowest), “time with family,” and “whole-person concerns” significantly declined over the last 1 or 2 months, with similar patterns observed in the proportion of scores categorized as “good to almost perfect.” Treatment preferences remained unchanged.

**Conclusions/Implications for Practice:**

Based on the findings, different aspects of EOL quality decline at different rates as patients approach death. In terms of the most poorly rated subdimensions, “symptoms and personal control” exhibited a substantial and accelerating decrease across the 6 months before death, while “death preparation” exhibited a gradual decline close to death only. Health care professionals should prioritize symptom management and death preparation for patients with terminal cancer by carefully assessing and promptly identifying their needs and developing effective, individualized care plans.

## Introduction

High-quality end-of-life (EOL) care and the alleviation of physical, psychological, and spiritual distress are of paramount importance to patients with terminal cancer (Institute of Medicine of the National Academies, 2015). However, as their disease progresses and worsens, these patients often experience not only multiple distressing symptoms (Teunissen et al., 2007) and mounting symptom severity (Henson et al., 2020) but also psychological distress related to their illness and poor prognosis (Chen et al., 2023). In a U.S.-based study, only 59.2% of bereaved family caregivers rated the quality of EOL care for their loved ones as excellent (Wachterman et al., 2016). Moreover, the EOL experiences of terminally ill cancer patients, as reported by bereaved family caregivers 6 months after patient death, revealed considerable scope for improvement (total EOL care score = 65.3, *SD* = 12.7, on a scale of 0–100), with the lowest scores observed in the domains of “transcendent” and “symptom management” (54.2 and 54.4, respectively; Hales et al., 2014). Notably, 16% to >50% of experiences in these domains were rated as poor (<40; Braun et al., 2014). These findings underscore the critical role of nursing care in addressing these issues through providing comprehensive support and symptom management to enhance EOL quality in patients with terminal cancer.

Understanding the perceptions of EOL quality and its associated domains among patients diagnosed with terminal cancer is essential to improving these patients’ EOL experience. EOL quality varies across countries and social resources (Finkelstein et al., 2022; Hoare et al., 2024). However, most prior research into EOL quality has failed to capture the values and preferences of dying patients because assessments are typically performed retrospectively, with data collected from the bereaved family members (Hoare et al., 2024). The lack of patient input not only contradicts the patient-centered nature of EOL care but may also yield results that do not accurately reflect patient perceptions of EOL quality, stemming from substantial differences in the subjective perceptions of patients and their family caregivers (Chen et al., 2024; Symmons et al., 2023). Moreover, data collected from these caregivers, all of whom were contacted between 1 and 8 months after the patient's death (Hoare et al., 2024), face significant risks of recall bias (Colombo et al., 2020), the effects of bereavement (Oechsle et al., 2020; Skantharajah et al., 2022), and other confounding factors (Hoare et al., 2024; Symmons et al., 2023).

Moreover, numerous cross-sectional studies have been conducted (Frey et al., 2020; Hoare et al., 2024) to explore EOL quality during the patient’s last 3 months. Of these, most have focused on the last month or week of patient life (Hoare et al., 2024), and their study designs do not consider potential variations in trends or the magnitudes of changes in EOL quality or associated domains as death approaches. The few related studies conducted using a prospective and longitudinal research design (Heyland et al., 2009; Lee & Yun, 2018) either depended on caregiver reports (Lee & Yun, 2018) or used small samples consisting of a mixture of patients with and without cancer that were followed up until patient death (Heyland et al., 2009). Therefore, to address these shortcomings, this study was designed to investigate the self-perceived changes of patients with cancer in terms of EOL quality and associated domains during their last 6 months of life. The following research question was asked: How does total EOL quality and its associated domains change during the last 6 months of life in patients with cancer?

## Methods

### Setting and Sample

This longitudinal prospective study was conducted on a convenience sample recruited from the oncology clinics/units of two medical centers in northern Taiwan, with data collected in January 2020 and April 2023 and followed up through July 2023. The participants were all adults with terminal cancer who were able to communicate with interviewers, showed advanced and metastatic cancer, were unresponsive to repeated curative chemotherapy/immunotherapy (Chen et al., 2022), and were clinically determined as incurable by their primary oncologist. The exclusion criteria were the following: (a) physician-diagnosed with a severe mental disorder (e.g., schizophrenia), (b) currently enrolled in another palliative care-related research study, (c) having no primary caregivers, (d) having a physical weakness that prevents participation, and (e) declining to provide informed consent. Patients with terminal cancer diagnoses were identified and referred to the researchers by primary physicians working at the targeted medical centers. After verifying eligibility, the data collectors invited these patients and their primary caregivers to participate.

The sample size required to detect changes in EOL quality was 156–241 based on a published effect size (Cohen’s *d*) of 0.18–0.23 (Lee & Yun, 2018), with 80% power and a .05 significance level, considering the number of repeated measures (median = 3; Chen et al., 2019), a 20% attrition rate (Tang et al., 2018), and adjusting for covariates.

The institutional review boards of the targeted medical centers approved the research protocol (Taipei Veterans General Hospital, 2019-11-003A, and National Taiwan University Hospital, 202111064RINC). While the original study focused on both patients and their primary caregivers, only the primary outcomes of the patients are reported here.

### Measures

#### Outcome Variable: EOL Quality

EOL quality was measured using the 31-item Quality of Dying and Death (QODD) questionnaire, which is designed to capture the quality of the dying and death experience across the following six main domains: symptoms and personal care, treatment preferences, time with family, whole-person concerns, death preparation, and moment of death (Patrick et al., 2001). The QODD was chosen for this study because it is widely used, offers reliability and good construct validity (Curtis et al., 2002), captures the respondent’s subjective experiences, and covers essential aspects of EOL quality (Dy et al., 2008; Singer et al., 1999). The construct validity of this questionnaire is supported by significant associations between higher QODD scores and key indicators of high-quality EOL care, including preferred place of death, lower symptom burden, and greater family satisfaction with health care communication (Curtis et al., 2002). However, some items, including moment of death (3 items), funeral arrangements, saying goodbye, spending time with pets, having the means to end life, and being able to laugh and smile, were removed, and the item spending time with spouse/partner, children, family, and friends was combined into spending time with family and friends to ensure the appropriateness of repeated assessments and cultural considerations. Thus, the final version of the modified 21-item QODD included symptoms and personal care (6 items), death preparation (8 items), time with family (2 items), treatment preferences (2 items), and whole-person concerns (3 items). Phrasing referring to “her/his” aimed at bereaved family respondents was modified to “your” for the sake of patients. The modified QODD demonstrated strong content validity, with a content validity index (CVI) of .98 based on ratings from five oncology and palliative care experts. As all items reported a CVI ≥.8, no further modifications were made. Reliability was supported by a Cronbach’s alpha of .90 (Chen et al., 2024).

The participants were asked to score their experiences throughout the EOL process on a 0–10 scale, with 0 indicating a negative experience and 10 showing an almost perfect experience. The scores for the 21 items were averaged and then multiplied by 10 to generate a total QODD score (ranging from 0 to 100). This transformation recalibrated the total score to a range commonly used in health-related quality measures, enhancing interpretation and facilitating cross-study comparability (Curtis et al., 2002; Moshina et al., 2021). To elucidate the EOL quality experience of the participants, the scores were further grouped into “good to almost perfect” (≥70) and “not good to unsatisfactory” (<70; Hales et al., 2014).

#### Covariates

Covariates known to influence EOL quality, including patient demographics (e.g., age, gender, level of education; Braun et al., 2014; Curtis et al., 2002; Hales et al., 2014) and clinical characteristics (e.g., disease diagnosis and location of care; Braun et al., 2014; Heyland et al., 2009), were adjusted for in this study.

### Data Collection

During the study period, trained and experienced oncology nurses explained the study to eligible patients and their caregivers while they were at the medical center, invited them to participate, and obtained signed informed consent. Subsequently, these same nurses interviewed the participants during hospitalization or clinic visits (baseline assessment), recording demographics, clinical characteristics, and the outcome variables. Follow-up assessments were conducted approximately every 3–4 weeks thereafter (Chen et al., 2022) during outpatient visits or rehospitalization to collect data on the outcome variables and the location of care provision until patients withdrew from the study or died.

### Data Analysis

Participant demographics, clinical characteristics, and EOL quality in various domains were described using descriptive statistics. To explore changes in EOL quality during their last 6 months of life, time proximity to death (the period between death and assessment) was categorized as 1–30, 31–60, 61–90, and 91–180 days. The changes in EOL quality over these last 6 months were examined using multivariate linear regression analyses for continuous variables (change in mean scores) and logistic regression analyses for categorical variables (odds of scoring ≥70, i.e., “good or almost perfect”) using generalized estimating equations (GEE) after controlling for covariates such as age, gender, educational level, disease diagnosis, and location of care provision. The GEE approach was used in this study to address variations in the number and timing of repeated measures across participants. It provides robust standard error estimates to account for within-subject correlations of QODD scores during the follow-up period (Liang & Zeger, 1986). Also, GEE enables the inclusion of incomplete data attributable to disease progression by treating each participant’s observations as independent clusters, enabling valid estimation despite variations in follow-up duration. Under the assumption that missing data occur at random, GEE utilizes all available observations rather than excluding participants with missing values, thereby preserving the sample size and minimizing bias (Liang & Zeger, 1986). The regression parameters for each independent variable were exponentiated to obtain the adjusted odds ratio (a*OR*) with a 95% CI, and a *p*-value of < .05 was considered statistically significant. IBM SPSS Statistics, 22.0 (IBM Corp., Armonk, NY, USA), was used to perform all statistical analyses.

## Results

### Sample Characteristics

Of the 403 patients with terminal cancer identified and referred by primary physicians, 50 were excluded due to the absence of a family caregiver, resulting in 353 potential participants. Of these, 280 were enrolled as participants (participation rate = 79.3%). The primary reasons for nonenrollment included family caregiver refusal (*n* = 3) and patient inability to participate (*n* = 70) due to physical weakness or lack of interest. The data from 51 of the participants were subsequently excluded from the final analysis for the following reasons: still alive at the end of the study period and thus lacked a recorded time of death for retrospective analysis (*n* = 50), and data were collected more than 6 months before death (*n* = 1). Excluding the data from these individuals ensured a focused analysis of quality changes in the final months of life and enhanced the accuracy and consistency of findings. Therefore, the final sample consisted of 229 participants, all of whom died during the study period. Detailed baseline demographics, clinical characteristics, and QODD for the final sample are shown in Table 1. Mean age was 60.14 (*SD* = 11.46, range 25.79–92.18) years, with nearly three-quarters (72.5%) over 55 years old. Most were male (62.4%), married (77.7%), had a high school or lower level of education (63.8%), and had sufficient financial resources (95.2%). The most common diagnosis was liver cancer (42.8%), followed by lung (21.0%) and gastrointestinal (10.5%) cancers. Most (72.9%) presented with metastatic disease at diagnosis. The average time from diagnosis to enrollment was 35.16 months (*SD* = 44.86; range = 1–290 months; median = 19.2 months), and most (71.2%) had comorbid chronic conditions (Table 1).

**Table 1:** Participant Demographics and Clinical Characteristics (*N* = 229)

Variable	*n* (%)
Age (years, mean and *SD*)	60.14 (11.46)
Median (range)	61.57 (25.79–92.18)
Gender	
Male	143 (62.4)
Female	86 (37.6)
Marital status	
Married	178 (77.7)
Not married	51 (22.3)
Educational level	
> High school	83 (36.2)
≤ High school	146 (63.8)
Financial status ^a^	
Adequate	199 (95.2)
Inadequate	10 (4.8)
Cancer site	
Liver	98 (42.8)
Lung	48 (21.0)
Gastrointestinal	24 (10.5)
Breast	22 (9.6)
Others	37 (16.2)
Metastasis	
Yes	167 (72.9)
No	62 (27.1)
Chronic disease	
Yes	163 (71.2)
No	66 (28.8)
Postdiagnostic survival (months, mean and *SD*)	35.16 (44.86)
Median (range)	19.20 (1–290)
Postenrollment survival (days; mean and *SD*) ^b^	96.86 (62.16)
Median (range)	96 (1–180)
QODD score (mean and *SD*) ^b^	
Symptoms and personal control	64.56 (14.12)
Death preparation	68.41 (9.21)
Time with family	75.22 (8.70)
Treatment preference	75.55 (10.43)
Whole-person concerns	70.54 (11.73)
QODD total score	68.91 (8.71)
QODD score: good to almost perfect (%) ^b^, ^c^	
Symptoms and personal control	104 (45.4)
Death preparation	118 (51.5)
Time with family	196 (85.6)
Treatment preparation	189 (83.3)
Whole-person concerns	156 (68.1)
QODD total score	125 (54.6)

*Note.* QODD = Quality of Dying and Death.

^a^ missing data; ^b^ Time-varying variables in the first assessment within participants’ last six months; ^c^ Frequency of QODD scale domain scores in “good to almost perfect” range (≥70).

The mean duration between study enrollment and death was 96.86 days (*SD* = 62.16; range = 1–180 days; median = 96 days). The survival time ranges after enrollment, i.e., 1–30, 31–60, 61–90, and 91–180 days, were 24.0%, 13.5%, 10.9%, and 51.5%, respectively. Approximately half of the participants (48.5%) survived for less than 3 months, and one-quarter (24.0%) died within 1 month of enrollment. The 173, 154, 121, and 118 participants in each time range, respectively, received 240, 198, 157, and 331 assessments. During the last 6 months of the study period, each participant completed an average of four assessments (*SD* = 2.40, median = 3, range = 1–9; 76.7% ≥2 assessments), with an average interval of 24.57 days between each assessment (*SD* = 11.52, median = 21, range = 7–69). The final assessment was conducted an average of 21.99 days before death (*SD* = 22.00, median = 16, range = 1–67 days).

### Overview of EOL Quality and Its Changes During the Last 6 Months

The initial assessment of EOL quality conducted showed a total score of 68.91 (*SD* = 8.71). In terms of mean initial subdomain scores, the highest were observed for “treatment preferences” and “time with family” (75.55 ± 10.43 and 75.22 ± 8.70, respectively), while the lowest were observed for “symptoms and personal control” and “death preparation” (64.56 ± 14.12 and 68.41 ± 9.21, respectively; Table 1). The EOL quality subdomains were similarly ranked in terms of mean score during the last month of life.

**Figure 1:**
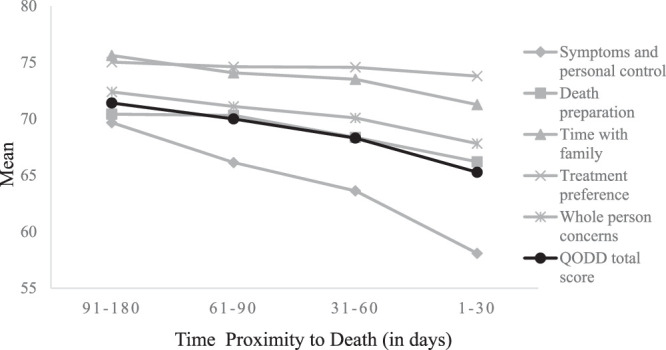
Changes in EOL Quality During Participants’ Last 6 Months of Life

During the last 6 months before death, total and subdomain scores show different declines as death approached (Figure 1). Changes in EOL quality during this period were examined by controlling for covariates, with the results indicating a significant decline in total EOL quality score during the last 2 months of life compared with 91–180 days before death (β [95% CI] = –2.51[–3.95, –1.06], *p* = .001; –5.13 [–6.78, –3.47], *p* < .001 for 31–60, 1–30 days before death, respectively; Table 2). In terms of the subdomain scores, the most pronounced changes were observed in “symptoms and personal control” over the 6-month period (mean ± *SD*, from 69.67 ± 12.02 to 58.09 ± 15.96); “symptoms and personal control” declined significantly during the final three months compared with 91–180 days before death (β [95% CI]= –3.31 [–5.48, –1.14], *p* = .003; –4.96 [–7.05, –2.87], *p* < .001; –9.57 [–12.27, –6.88], *p* < .001, for 61–90, 31–60, and 1–30 days before death, respectively); “death preparation” and “time with family” significantly declined in the last 2 months (β [95% CI] = –1.66 [–3.26, –0.07], *p* = .041; –3.42 [–5.13, –1.72], *p* < .001; and –1.82 [–3.37, –0.26], *p* = .022; –4.00 [–5.78, –2.23], *p* < .001, for 31–60 and 1–30 days before death, respectively); and “whole-person concerns” declined significantly only in the last month before death (β [95% CI] = –3.78 [–5.79, –1.77], *p* < .001) compared with 91–180 days before death. Scores in the subdomain “treatment preferences” remained constant across the study period (Table 2).

**Table 2:** Multivariate Analysis of Changes in EOL Quality Scores Over the Last 6 Months of Life (*N* = 229)

Variable	QODD Total Score				Symptoms and Personal Control				Death Preparation			
	Mean (*SD*)	β	95% CI	*p*	Mean (*SD*)	β	95% CI	*p*	Mean (*SD*)	β	95% CI	*p*
Time proximity to death (in days)												
1–30	65.27 (10.04)	−5.13	[-6.78, -3.47]	<.001	58.09 (15.96)	−9.57	[−12.27, −6.88]	<.001	66.20 (10.22)	−3.42	[−5.13, −1.72]	<.001
31–60	68.30 (8.36)	−2.51	[−3.95, −1.06]	.001	63.62 (13.05)	−4.96	[−7.05, −2.87]	<.001	68.36 (8.52)	−1.66	[−3.26, −0.07]	.041
61–90	70.00 (8.60)	−1.11	[−2.65, 0.43]	.157	66.13 (13.01)	−3.31	[−5.48, −1.14]	.003	70.31 (8.11)	0.29	[−1.28, 1.86]	.722
91–180	71.41 (7.56)	Ref			69.67 (12.02)	Ref			70.42 (7.81)	Ref		
Age	—	−0.02	[−0.09, 0.05]	.550	—	−0.07	[−0.19, 0.05]	.232	—	0.01	[−0.06, 0.08]	.800
Gender												
Male	69.17 (9.14)	0.19	[−1.41, 1.80]	.812	65.03 (14.84)	−0.48	[−2.90, 1.95]	.701	69.27 (8.63)	0.88	[−0.76, 2.53]	.293
Female	68.45 (8.54)	Ref			64.29 (13.08)	Ref			68.12 (9.22)	Ref		
Educational level												
> High school	69.29 (8.65)	0.70	[−0.96, 2.37]	.407	65.07 (14.90)	0.17	[−2.54, 2.87]	.903	68.99 (8.60)	0.46	[−1.20, 2.13]	.584
≤ High school	68.70 (9.09)	Ref			64.60 (13.86)	Ref			68.90 (9.01)	Ref		
Cancer site												
Lung	67.94 (7.92)	−3.63	[−5.35, −1.92]	<.001	63.90 (13.03)	−4.88	[−7.69, −2.08]	.001	68.70 (7.08)	−1.54	[−3.24, 0.16]	.076
Breast	69.45 (8.92)	−1.90	[−5.04, 1.23]	.234	65.42 (14.77)	−3.16	[−7.81, 1.50]	.184	68.14 (10.16)	−1.33	[−4.90, 2.25]	.468
Gastrointestinal	68.71 (7.93)	−3.36	[−6.02, −0.70]	.013	66.03 (14.40)	−3.79	[−8.12, 0.53]	.085	69.01 (6.40)	−1.47	[−3.72, 0.77]	.198
Others	66.05 (9.57)	−4.76	[−7.06, −2.46]	<.001	59.66 (16.18)	−7.66	[−11.48, −3.85]	<.001	66.91 (8.84)	−2.74	[−4.91, −0.56]	.014
Liver	70.88 (9.25)	Ref			67.20 (13.49)	Ref			70.00 (10.17)	Ref		
Care site												
Hospital	71.26 (7.11)	−3.09	[−4.19, −1.98]	<.001	60.16 (15.39)	−6.14	[−7.93, −4.35]	<.001	67.18 (9.72)	−2.14	[−3.29, −0.98]	<.001
Outpatient	66.59 (9.90)	Ref			69.42 (11.23)				70.57 (7.53)	Ref		
Variable	Time With Family				Treatment Preference				Whole-Person Concerns			
	Mean (*SD*)	β	95% CI	*p*	Mean (*SD*)	β	95% CI	*p*	Mean (*SD*)	β	95% CI	*p*
Time proximity to death (in days)												
1–30	71.26 (12.43)	−4.00	[−5.78, −2.23]	<.001	73.79 (10.51)	−1.64	[−3.50, 0.22]	.084	67.81 (11.89)	−3.78	[−5.79, −1.77]	<.001
31–60	73.51 (9.42)	−1.82	[−3.37, −0.26]	.022	74.57 (8.50)	−0.58	[−2.13, 0.96]	.462	70.08 (11.28)	−1.59	[−3.50, 0.32]	.103
61–90	74.08 (8.39)	−1.20	[−2.84, 0.43]	.150	74.62 (8.80)	−0.38	[−2.04, 1.28]	.656	71.10 (11.59)	−1.22	[−3.03, 0.58]	.184
91–180	75.62 (7.40)	Ref			75.03 (8.61)	Ref			72.39 (10.40)	Ref		
Age	—	0.04	[−0.04, 0.12]	.324	—	−0.01	[−0.09, 0.06]	.730	—	−0.04	[−0.13, 0.06]	.445
Gender												
Male	73.88 (9.77)	0.13	[−1.82, 2.09]	.896	74.81 (9.16)	0.38	[−1.37, 2.14]	.669	70.35 (11.78)	−0.87	[−3.13, 1.40]	.453
Female	73.60 (9.43)	Ref			74.04 (9.11)	Ref			70.75 (10.42)	Ref		
Educational level												
> High school	74.30 (8.97)	1.29	[−0.46, 3.04]	.148	75.15 (8.28)	1.46	[−0.18, 3.09]	.080	71.29 (10.75)	1.36	[−0.79, 3.51]	.216
≤ High school	73.80 (10.12)	Ref			74.19 (9.60)	Ref			70.02 (11.62)	Ref		
Cancer site												
Lung	72.82 (9.32)	−2.23	[−4.23, −0.22]	.029	72.10 (7.89)	−5.43	[−7.16, −3.69]	<.001	67.95 (10.92)	−7.14	[−9.48, −4.81]	<.001
Breast	74.43 (8.59)	−0.38	[−3.47, 2.71]	.811	75.19 (9.66)	−2.76	[−5.91, 0.39]	.086	73.88 (9.10)	−1.94	[−5.33, 1.45]	.263
Gastrointestinal	74.02 (7.89)	−0.59	[−3.19, 2.02]	.660	71.34 (8.02)	−6.52	[−9.24, −3.81]	<.001	68.04 (10.64)	−7.66	[−11.22, −4.10]	<.001
Others	73.29 (10.28)	−1.31	[−3.78, 1.15]	.296	73.88 (9.04)	−3.58	[−5.95, −1.21]	.003	66.58 (13.41)	−7.88	[−11.53, −4.22]	<.001
Liver	74.52 (10.26)	Ref			77.51 (9.42)	Ref			74.11 (9.96)	Ref		
Care site												
Hospital	72.91 (11.23)	−0.94	[−2.26, 0.38]	.162	74.71 (11.03)	0.22	[−0.96, 1.41]	.712	68.64 (12.92)	−2.95	[−4.36, −1.55]	< .001
Outpatient	74.65 (7.64)	Ref			74.37 (6.77)	Ref			72.36 (9.07)	Ref		

*Note.* Multivariate linear regression with the generalized estimating equations was conducted.

At 1–30, 31–60, 61–90, and 91–180 days before death, 173, 154, 121, and 118 participants collectively received 240, 198, 157, and 331 assessments, respectively. Each participant completed up to nine assessments over the study period, with varying numbers of assessments varying across different time intervals. QODD = Quality of Dying and Death; CI = confidence interval; Ref = reference.

### Changes in the Frequency Distribution of EOL Quality

The scores were grouped into two categories, with those ≥70 indicating “good to almost perfect” EOL quality. At the initial assessment, 54.6% of participants rated their total EOL quality within the “good to almost perfect” range (Table 1). In terms of subdomains, experience of time with family and treatment preferences earned the highest percentages of “good to almost perfect” scores (85.6% and 83.3%, respectively), while “symptoms and personal control” and “death preparation” earned the lowest (45.4% and 51.5%, respectively; Table 1). Thus, notably, roughly half of the participants initially reported a high level of satisfaction in even the lowest-scoring category.

Total and subdomain scores reaching the “good to almost perfect” category showed varying rates of decline as death approached (Table 3). The proportion of EOL quality total scores in the “good to almost perfect” category decreased from 68.6% (91–180 days) to 34.6% (1–30 days). Multivariate logistic regression revealed a significant decrease in total score during the last 2 months before death compared with the 91–180 day period (a*OR* [95% CI] = 0.51 [0.36–0.74], *p* < .001; 0.27 [0.18–0.40], *p* < .001 for 31–60 and 1–30 days, respectively; Table 3). In terms of subdomain scores reaching the “good to almost perfect” category, “symptoms and personal control” showed the most substantial decline (61.9%–26.7%), followed by “death preparation” (65.3%–39.6%), “whole-person concerns” (79.2%–53.3%), and “time with family” (89.1%–70.3%) over the last 6-month period, while “treatment preference” (86.1%–79.5%) showed only a minor change. Compared with 91–180 days before death, “symptoms and personal control” and “whole-person concerns” exhibited a significant decline during the last 3 months (a*OR* [95% CI] = 0.66 [0.45–0.98], *p* = .038; 0.42 [0.29–0.59], *p* < .001; 0.26 [0.17–0.39], *p* < .001; and 0.55 [0.35–0.84], *p* = .006; 0.62 [0.42–0.91], *p* = .016; 0.32 [0.20–0.51], *p* < .001 for 61–90, 31–60, and 1–30 days before death, respectively), while “death preparation” and “time with family” showed a significant decline during the last 2 months (0.54 [0.38–0.78], *p* = .001; 0.40 [0.28–0.58], *p* < .001; and 0.53 [0.30–0.92], *p* = .024; 0.33 [0.20–0.55], *p* < .001, for 31–60, and 1–30 days, respectively). However, treatment preferences did not significantly change over time (Table 3).

**Table 3:** Multivariate Analysis of Changes in “Good to Almost Perfect” EOL Quality Scores Over the Last 6 Months of Life ^a^ (*N*= 229)

Variable	QODD Total Score			Variable			QODD Total Score		
	%	a*OR* (95% CI)	*p*	%	a*OR* (95% CI)	*p*	%	a*OR* (95% CI)	*p*
Time proximity to death (in days)									
1–30	34.6	0.27 [0.18, 0.40]	<.001	26.7	0.26 (0.17, 0.39)	<.001	39.6	0.40 [0.28, 0.58]	<.001
31–60	51.5	0.51 [0.36, 0.74]	<.001	37.9	0.42 (0.29, 0.59)	<.001	48.5	0.54 [0.38, 0.78]	.001
61–90	61.1	0.77 [0.53, 1.11]	.157	50.3	0.66 (0.45, 0.98)	.038	56.1	0.72 [0.50, 1.04]	.077
91–180	68.6	Ref		61.9	Ref		65.3	Ref	
Age		1.00 [0.98, 1.02]	.872		0.99 (0.97, 1.01)	.456		1.00 [0.98, 1.02]	.956
Gender									
Male	57.5	1.25 [0.83, 1.90]	.285	48.5	1.33 (0.88, 2.00)	.176	55.6	1.16 [0.78, 1.74]	.463
Female	50.0	Ref		40.4	Ref		49.4	Ref	
Educational level									
> High school	55.1	1.13 [0.75, 1.71]	.566	47.2	1.20 (0.78, 1.84)	.401	54.0	1.10 [0.75, 1.63]	.614
≤ High school	54.7	Ref		44.8	Ref		53.2	Ref	
Cancer site									
Lung	46.6	0.31 [0.19, 0.49]	<.001	39.8	0.40 (0.25, 0.64)	<.001	48.1	0.46 [0.31, 0.70]	<.001
Breast	58.2	0.65 [0.32, 1.33]	.242	45.6	0.67 (0.32, 1.38)	.277	53.2	0.70 [0.34, 1.44]	.333
Gastrointestinal	48.5	0.31 [0.16, 0.59]	<.001	46.4	0.45 (0.23, 0.87)	.018	53.6	0.55 [0.31, 0.97]	.038
Other	41.6	0.31 [0.18, 0.54]	<.001	32.2	0.34 (0.19, 0.61)	<.001	40.3	0.38 [0.23, 0.63]	< .001
Liver	68.2	Ref		56.1	Ref		63.5	Ref	
Care site									
Hospital	43.4	0.47 (0.35, 0.64]	<.001	31.2	0.40 (0.29, 0.56)	<.001	44.3	0.58[0.43, 0.78]	<.001
Outpatient	66.4	Ref		60.3	Ref		62.7	Ref	
	Time With Family			Treatment Preference			Whole-Person Concerns		
Variable	%	*AOR* (95% CI)	*p*	%	*AOR* (95% CI)	*p*	%	*AOR* (95% CI)	*p*
Time proximity to death (in days)									
1–30	70.3	0.33 [0.20, 0.55]	<.001	79.5	0.71 [0.42, 1.19]	.190	53.3	0.32 [0.20, 0.51]	<.001
31–60	80.3	0.53 [0.30, 0.92]	.024	82.7	0.82 [0.50, 1.36]	.443	68.2	0.62 [0.42, 0.91]	.016
61–90	81.5	0.59 [0.34, 1.01]	.053	86.0	1.00 [0.57, 1.76]	.990	68.8	0.55 [0.35, 0.84]	.006
91–180	89.1	Ref		86.1	Ref		79.2	Ref	
Age		1.02 [1.00, 1.04]	.064		1.00 [0.98, 1.02]	.917		0.99 [0.97, 1.01]	.473
Gender									
Male	81.4	0.92 [0.58, 1.44]	.702	84.7	1.13 [0.71, 1.81]	.605	68.3	0.84 [0.53, 1.32]	.450
Female	80.6	Ref		81.7	Ref		68.5	Ref	
Educational level									
> High school	82.4	1.38 [0.89, 2.15]	.154	86.4	1.56 [0.98, 2.49]	.063	70.1	1.19 [0.74, 1.91]	.466
≤ High school	80.3	Ref		82.0	Ref		67.4	Ref	
Cancer site									
Lung	76.9	0.52 [0.32, 0.84]	.008	78.8	0.34 [0.20, 0.56]	<.001	57.6	0.18 [0.11, 0.30]	<.001
Breast	81.0	0.75 [0.33, 1.71]	.499	83.5	0.50 [0.21, 1.19]	.117	81.0	0.61 [0.27, 1.37]	.232
Gastrointestinal	84.5	1.07 [0.57, 2.00]	.830	77.3	0.29 [0.13, 0.63]	.002	57.7	0.17 [0.09, 0.34]	<.001
Others	80.5	0.82 [0.46, 1.49]	.525	80.3	0.42 [0.23, 0.77]	.005	53.7	0.20 [0.11, 0.37]	<.001
Liver	83.6	Ref		90.8	Ref		83.4	Ref	
Care site									
Hospital	75.9	0.61 [0.42, 0.90]	.012	79.9	0.58 [0.38, 0.88]	.011	58.3	0.39 [0.28, 0.54]	<.001
Outpatient	86.3	Ref		87.4	Ref		78.5	Ref	

*Note.* Multivariate logistic regression with the generalized estimating equations was conducted.

At 1–30, 31–60, 61–90, and 91–180 days before death, 173, 154, 121, and 118 participants, respectively, received 240, 198, 157, and 331 assessments. Each participant completed up to nine assessments over the study period, with varying numbers of assessments varying across different time intervals. QODD = Quality of Dying and Death; a*OR* = adjusted odds ratio; CI = confidence interval; Ref = reference.

^a^ Frequency of QODD scale domain scores in “good to almost perfect” range (≥70).

## Discussion

In this study, the EOL quality total score and subdomain scores showed varying rates of decline as death approached. The scores taken at baseline are similar to those found in prior comparable research settings (Hales et al., 2014; Mah et al., 2019), but higher than those observed in one report (Wang et al., 2021). In addition, changes in EOL quality total score declined significantly during the final 2 months of life, which aligns with a longitudinal study (Lee & Yun, 2018) and contrasts with another (Heyland et al., 2009) in which no significant changes were shown in EOL quality as death approached. Possible reasons for this discrepancy include the small sample size and inclusion of patients without cancer in the Heyland study. The results of this study include a decline in the total EOL quality score over the last 6 months of life and a notable drop of more than 30% in the proportion of patients experiencing a “good to almost perfect” EOL quality close to death. This highlights a substantial opportunity for improving EOL quality in this patient group. Efforts to enhance EOL quality should be applied consistently throughout the final months of life rather than intensely during the dying stages only.

With regard to subdomain scores, “symptoms and personal control” was associated with the lowest degree of satisfaction, followed by “death preparation,” while “time with family” and “treatment preferences” were rated as the most satisfactory. During the last 6 months of life, mean “symptoms and personal control” scores declined significantly, especially as participants experienced accelerated deterioration close to death, along with the proportion of scores in the “good to almost perfect” category. Furthermore, mean scores for the “death preparation,” “time with family,” and “whole-person concerns” subdimensions dropped significantly during the last 1–2 months before death, with the proportion of “good to almost perfect” scores decreasing by approximately 20% during the last 6 months of life. This ranking of subdomain scores aligns with other studies (Mah et al., 2019) but is inconsistent with one (Heyland et al., 2009) that showed similarities among all domains and no significant change in satisfaction with illness management as death approached.

The results of this study indicate “symptoms and personal control” was the least satisfactory subdomain, exhibiting a substantial decline, with the proportion of patients rating this domain as “good to almost perfect” decreasing dramatically from 61.9% to 26.7% during the last 6 months of life. This decline may be attributed to the increasing occurrence of multiple distressing symptoms, as well as the “terminal drop” phenomenon (Versluis et al., 2024), wherein cancer patients experience rapidly worsening physiological symptoms in the last months of life (Henson et al., 2020). Therefore, developing optimal interventions that address symptoms and personal control is paramount and urgent to enhance EOL quality and identify disease-related distress as early as possible.

The “death preparation” subdomain (the second-lowest score) and the proportion of assessment items categorized as “good to almost perfect” (decreasing from 65.3% to 39.6% over the last 6 months) demonstrated a significant decline in the last 2 months compared with 91–180 days before death. This finding is consistent with Tang et al. (2019), who also identified a decline in emotional preparedness for death. The deterioration of physical symptoms in cancer patients can impact their preparedness for death, including increased fear of death, concerns over unfinished business, and deteriorating spiritual well-being (Miller et al., 2022). Moreover, health care professionals’ failure to address the spiritual discomfort of their patients is a common insufficiency in current clinical practice due to insufficient training and preparation, as well as time/resource constraints leading to a focus on addressing physical symptoms to the neglect of the spiritual dimension of care (Chiang et al., 2020; Zumstein-Shaha et al., 2020). Furthermore, patient reluctance to discuss death-related issues or their unexpressed needs for spiritual exploration, as shown in the authors’ previous report (Chen et al., 2022), suggests a considerable proportion of patients with cancer remain unaware of/unwilling to acknowledge their disease, making it challenging for health care professionals to identify and respond to their underlying needs.

In this study, the mean score for “whole-person concerns” fell in the middle of the five domains and significantly declined in the last month before death, with the proportions of patients rating this domain as “good to almost perfect” decreasing from 79.2% to 53.3% during the last 6 months. Late-stage cancer may lead to a significant decline in dignity and sense of meaning, as worsening symptoms increase dependence and reduce autonomy, thereby compromising personal values (Dorr et al., 2023). Although effective symptom management can enhance a sense of control, life review may help individuals rediscover meaning and restore dignity, ultimately improving psychological well-being and overall quality of life (Huang et al., 2020).

In contrast, “time with family” and “treatment preference” earned the highest mean EOL quality subdimension scores in this study. The “time with family” score significantly declined during the last 2 months of life, with a similar trend observed in “good to almost perfect” category scores. However, this trend differs from another study (Lee & Yun, 2018) that found the lowest mean levels of satisfaction to be in “family relationships” and no significant change over a 3-month period. The higher satisfaction with spending time with family found in this study may be attributable to the inclusion of patients and their primary caregivers, helping the participants feel cared for and supported by their families. Moreover, a significant decline was observed in the evaluation of “time with family” over the last 2 months of life, which may stem not only from worsening symptoms and emotional distress (Oppegaard et al., 2022) but also from mobility-related social isolation and caregiver strain due to personal or patient-related factors (Yang et al., 2023). These challenges may result in reduced family interaction and lower satisfaction with this domain.

The mean score for “treatment preference” was the highest among all of the subdomains, with the highest proportion of participants ranking it as “good to almost perfect” (79.5%–86.1%). Moreover, this rating did not significantly change as death approached. This finding is consistent with a previous longitudinal study (Heyland et al., 2009), which showed that patient satisfaction with communication and decision-making remained stable as death approached. The favorable ratings in this domain may be ascribed to the fact that most of the participants (90.6%–92.1%) had discussed their EOL care wishes with their physicians during the last 6 months of life, and that 20.2%–46.3% had discussed with their physicians about receiving life-sustaining treatments (data not shown), which may reflect individual patient needs or readiness to engage in such discussions. However, over 50% of the participants did not discuss preferences regarding life-sustaining treatments with their physicians during the last month of life, potentially increasing the risk of receiving aggressive treatments at EOL (Knutzen et al., 2021). Therefore, health care professionals should work to communicate continuously with their patients to ensure their preferences and wishes are respected and followed.

### Limitations

Several limitations of this study should be noted. The convenience sample recruited from two medical centers in Taiwan limits the generalizability of the findings to broader national and international populations. In the authors’ original study, patients and their primary caregivers were recruited together. Therefore, the findings may not be generalizable to patients who lack a primary caregiver. In this study, 24.0% of the participants were recruited in their last month of life, and 17.5% were assessed only once, which may limit the observation and tracking of individual patient changes over time, potentially compromising study validity. Future research should collaborate with oncology teams to screen eligible patients proactively in outpatient clinics and hospital wards using prognosis-predicting tools (e.g., Supportive and Palliative Care Indicators Tool [SPICT], Necesidades Paliativas [NECPAL]) for earlier identification (ElMokhallalati et al., 2020). In addition, flexible follow-up assessments can improve data completeness and retention by adapting to patient conditions. The original focus of the QODD instrument was on bereaved family members and their retrospective evaluation of the quality of their loved ones’ lives during their final week or month of life. Thus, in this study, a longitudinal and prospective design was adopted, and a modified QODD (in which unsuitable domains and subitems either designed for long-term follow-up or reflective of nonrelevant cultural practices had been removed) was used to assess patients’ subjective perceptions of their EOL quality. Finally, the validity and reliability measures used in this study warrant further validation for use with terminally ill cancer patients.

### Conclusions and Clinical Implications

The findings of this study confirm that EOL quality total and subdomain scores change over time in patients with terminal cancer during their last 6 months of life. In terms of the EOL quality subdomains, “time with family” and “treatment preferences” were associated with the highest mean satisfaction scores. The former dropped significantly only during the last 2 months of life, while “treatment preferences” registered a slight decline only. “Symptoms and personal control” and “death preparation” were identified as the poorest-performing domains, with related scores decreasing significantly in the last 3 and 2 months of life, respectively. In light of these findings, health care professionals should prioritize symptom management and enhance the functional independence of patients as much as possible by carefully assessing their symptom distress, understanding their expectations regarding symptom control, promoting communication and collaboration, and developing and implementing evidence-based care guidelines for symptom control. Furthermore, health care professionals should facilitate death preparation in patients with cancer through the timely identification of death-related issues tailored to their needs, responding to them with sensitivity and empathy, providing emotional support, and making appropriate referrals to related resources as needed. These strategies and related measures can optimize EOL quality and promote quality of life in patients with terminal cancer.
